# Maternal and Fetal Outcomes in Pregnant Women With Lung Cancer: A Population‐Based Study on 9 Million Pregnancies and 40 Cases of Lung Cancer

**DOI:** 10.1111/1471-0528.18090

**Published:** 2025-02-11

**Authors:** Samantha Jacobson, Ahmad Badeghiesh, Haitham Baghlaf, Noah Margolese, Michael H. Dahan

**Affiliations:** ^1^ Faculty of Medicine University of Sherbrooke Sherbrooke Canada; ^2^ Department of Obstetrics and Gynecology King Abdulaziz University Rabigh Saudi Arabia; ^3^ Department of Obstetrics and Gynecology University of Tabuk Tabuk Saudi Arabia; ^4^ Department of Obstetrics and Gynecology McGill University Health Centre Montreal Canada

**Keywords:** haematology: coagulation, maternal mortality, obstetric haemorrhage

Lung cancer during pregnancy is exceedingly rare, with only 93 cases reported in the literature from 1953 to 2024 [1]. It carries the highest mortality rate of cancers in pregnancy (64.3%) [2] and increases risks of placental abnomalities, preterm delivery and low birth weight, with delayed diagnosis often due to nonspecific symptoms [1, 2]. Using a 9‐million patient database, we identified 40 additional cases, analysed independently in this study, bringing the total to 133. The objective is to evaluate maternal and fetal outcomes using this dataset, comparing pregnancies with and without lung cancer.

We conducted a retrospective population‐based study using data from the Health Care Cost and Utilisation Project‐Nationwide Inpatient Sample (HCUP‐NIS) database (2004–2014). Lung cancer cases were identified using the ICD‐9 code 162.x. The study group included pregnant woman with lung cancer, while all other deliveries were controls. Categorical variables were compared using chi‐squared tests, except when any cell frequency was less than 5, in which case Fisher's exact test was applied to ensure validity. Logistic regression analysed associations between lung cancer and maternal and fetal outcomes, estimating odds ratios (ORs) and 95% confidence intervals (CIs) adjusting for potential confounders, including maternal age, race, income, insurance type, smoking history, obesity, preexisting hypertension and diabetes.

Maternal characteristics are summarised in Table 1. Women with lung cancer were older, with significantly higher smoking rates (*p* = 0.01), chronic hypertension and pregestational diabetes (*p* < 0.0001). There were no significant differences in racial distribution, income quartiles, obesity, previous caesarean sections, thyroid disease or illicit drug. Medicaid and private insurance plans were more prevalent in the lung cancer group (*p* < 0.0001). Table 2 presents pregnancy, delivery and neonatal outcomes. Women with lung cancer had higher risks of placenta previa (OR: 5.67, 95% CI: 1.36–23.65, *p* = 0.017), abruptio placenta (OR: 4.99, 95% CI: 1.49–16.74, *p* = 0.009), operative vaginal delivery (OR: 4.88, 95% CI: 2.14–11.11, *p* < 0.001), transfusion (OR: 8.92, 95% CI: 3.28–24.28, *p* < 0.001), venous thromboembolism (VTE) (OR:21.83, 95% CI: 2.92–163.47, *p* < 0.001), disseminated intravascular coagulation (DIC) (OR: 8.45, 95% CI: 1.14–62.42, *p* = 0.04) and maternal death (OR: 195.02, 95% CI: 40.61–936.55, *p* < 0.001), though differences in spontaneous vaginal delivery became non‐significant after adjustment (OR: 0.58, 95% CI: 0.30–1.14, *p* = 0.112). Neonatal outcomes were similar between groups.

**TABLE 1:** Maternal characteristics of women with and without lung cancer who delivered between 2004 and 2014.

Characteristics	Lung cancer *N* = 40	Control *N* = 9 096 738 (%)	*p*
Age (years)			< 0.0001
< 25	< 11	3 455 851 (38%)	
25–34	< 11	4 299 894 (47.3%)	
≥ 35	27 (67.5%)	1 340 993 (14.7%)	
Race			0.09
White	27 (67.5%)	4 500 946 (49.5%)	
Black	< 11	1 690 049 (18.6%)	
Hispanic	< 11	2 034 092 (22.4%)	
Others	< 11	871 661 (9.6%)	
Income quartiles			0.56
Less than 39 000	< 11	2 218 131 (24.4%)	
$39 000‐47 999	12 (30%)	3 080 118 (33.9%)	
$48 000‐62 999	< 11	2 416 127 (26.6%)	
$63 000 or more	< 11	1 382 372 (15.2%)	
Insurance plan type			< 0.0001
Medicare	< 11	56 599 (0.62%)	
Medicaid	12 (30%)	3 882 764 (42.7%)	
Private including HMO	21 (52.5%)	4 606 952 (50.6%)	
Self‐pay	< 11	288 435 (3.2%)	
Other	< 11	261 998 (2.9%)	
Obesity (BMI ≥ 30 kg/m^2^)	< 11	324 174 (3.6%)	0.65
Previous CS	< 11	1 452 485 (16%)	0.55
Tobacco Smoking during pregnancy	< 11	443 584 (4.9%)	0.01
Chronic HTN	< 11	165 226 (1.8%)	< 0.0001
Pregestational DM	< 11	86 611 (0.95%)	< 0.0001
Thyroid disease	< 11	223 275 (2.5%)	0.08
Illicit drug use	< 11	125 618 (1.4%)	0.43

*Note*: Per the HCUP database regulations when less than 11 cases of an outcome occur it must be represented as < 11 to preserve patient anonymity.

Abbreviations: CS, caesarean section; DM, diabetes mellitus; HTN, hypertension.

**TABLE 2:** Pregnancy, delivery and neonatal outcomes for woman with and without lung cancer who delivered between 2004 and 2014.

Outcomes	Lung cancer *N* = 40	Control *N* = 9 096 738 (%)	Crude OR (95% CI)	*p*	Adjusted OR (95% CI)	*p* Adjusted
Pregnancy outcomes						
Pregnancy‐induced hypertension	< 11	673 747 (7.4%)	0.66 (0.16–2.73)	0.56	0.44 (0.10–1.91)	0.27
Gestational hypertension	< 11	301 606 (3.3%)	0.75 (0.10–5.44)	0.77	0.78 (0.11–5.66)	0.83
Preeclampsia	< 11	327 389 (3.6%)	0.69 (0.09–5.00)	0.71	0.63 (0.09–4.60)	0.65
GDM	< 11	523 191 (5.8%)	0.42 (0.06–3.06)	0.39	0.27 (0.04–1.94)	0.19
Placenta previa	< 11	49 980 (0.55%)	9.53 (2.30–39.42)	0.002	5.67 (1.36–23.65)	0.017
Delivery outcomes						
PPROM	< 11	103 617 (1.1%)	2.22 (0.31–16.20)	0.43	1.63 (0.22–11.98)	0.63
Preterm delivery	< 11	653 891 (7.2%)	1.44 (0.51–4.03)	0.49	0.90 (0.31–2.65)	0.85
Abruptio placenta	< 11	97 476 (1.1%)	7.49 (2.31–24.28)	0.001	4.99 (1.49–16.74)	0.009
Operative vaginal delivery	< 11	489 393 (5.4%)	4.40 (2.03–9.54)	< 0.0001	4.88 (2.14–11.11)	< 0.001
CS	17 (42.5%)	2 939 901 (32.3%)	1.55 (0.83–2.90)	0.17	0.94 (0.48–1.83)	0.85
Spontaneous vaginal delivery	15 (37.5%)	5 667 454 (62.3%)	0.36 (0.19–0.69)	0.002	0.58 (0.30–1.14)	0.112
PPH	< 11	263 964 (2.9%)	0.86 (0.12–6.24)	0.88	0.79 (0.11–5.75)	0.81
Transfusion	< 11	90 362 (0.99%)	14.58 (5.70–37.29)	< 0.0001	8.92 (3.28–24.28)	< 0.001
VTE	< 11	5309 (0.06%)	43.91 (6.03–319.65)	< 0.0001	21.83 (2.92–163.47)	0.003
DIC	< 11	18 243 (0.20%)	12.76 (1.75–92.88)	0.01	8.45 (1.14–62.42)	0.04
Maternal death	< 11	636 (0.007%)	752.74 (181.22–3126.75)	< 0.0001	195.02 (40.61–936.55)	< 0.001
Neonatal outcomes						
SGA	< 11	198 069 (2.2%)	0.89	1.15 (0.16–8.38)	0.85 (0.11–6.30)	0.87
IUFD	< 11	38 258 (0.42%)	0.07	6.07 (0.83–44.19)	3.25 (0.43–24.64)	0.25

*Note*: Per the HCUP database regulations when less than 11 cases of an outcome occur it must be represented as < 11 to preserve patient anonymity.

Abbreviations: CS, caesareancesarean section; DIC, disseminated intravascular coagulation; IUFD, intrauterine fetal demise; PPH, postpartum haemorrhagehemorrhage; PPROM, preterm premature rupture of membrane; SGA, small for gestational age; VTE, venous thromboembolism.

These findings align with existing literature indicating that pregnant women with lung cancer are older and present with comorbidities like chronic hypertension and pregestational diabetes [1], which can complicate pregnancy. Risk factors for placenta previa, including advanced maternal age and smoking [3], were also more common in the lung cancer group. The association with placenta previa could be due to vascular changes induced by cancer or its treatment; it is also possible that chemotherapy may impair placental development by inhibiting endothelial cell proliferation and angiogenic signalling via VEGF, reducing vascularisation [4]. However, we recognise that this finding may have been incidental rather than causally related. Increased incidence of abruptio placenta, VTE and DIC could be attributed to the hypercoagulable state in pregnancy as it increases clotting factors and promotes thrombosis [5]. This is further compounded by cancer‐related factors including venous stasis, tumour‐mediated release of procoagulant tissue factor and inflammatory cytokines like TNF‐alpha and PAI‐1 [5]. Abruptio placenta and placenta previa are known triggers for DIC [6], and cancer is associated with vascular placental [6] and coagulation abnormalities, potentially necessitating operative delivery and/or transfusion and sometimes resulting to maternal death. Although, the cancer itself is the most likely cause of the maternal death [2], delays in cancer treatment due to concerns about potential fetal harm can exacerbate outcomes.

Limitations include the retrospective design and reliance on the HCUP‐NIS database, which lacks granular clinical details such as cancer staging, treatment regimens and smoking history. This limits analysis of cancer treatments impact on pregnancy outcomes. ORs were calculated based on the specific number of cases and controls within each group, with outcomes involving fewer than 11 cases represented as < 11 in accordance with HCUP database regulations to maintain patient anonymity. The study period (2004–2014) reflects ICD‐9 use, as ICD‐10 codes, introduced in 2015, are not directly comparable. This may affect the generalisability of findings to more recent advancements in cancer treatment and prenatal care. The rarity of lung cancer during pregnancy resulted in a small sample size, reducing statistical power and increasing the risk of false‐positive results due to multiple comparisons. While we conducted a matched analysis to address confounding, the limited power of this approach failed to detect significant differences, even for outcomes like maternal death. While we acknowledge overfitting as a potential limitation, our unmatched analysis offers more robust insights, despite the inherent challenges of studying rare conditions.

By analysing 40 cases, this study quantified risks such as VTE (*p* < 0.001) and DIC (*p* = 0.04) and offered new insights into the association between lung cancer and placental complications. These findings enhance the understanding of specific risks and support the development of tailored antenatal care strategies for this high‐risk population, including recommendations for referral to high‐risk units and structured follow‐up of these pregnancies.

## Author Contributions

Samantha Jacobson: Writing – review & editing, Writing – original draft, Data curation. Ahmad Badeghiesh: Investigation, Formal analysis. Haitham Baghlaf: Investigation, Formal analysis. Noah Margolese: Writing – original draft. Michael H Dahan: Writing – review & editing, Supervision, Methodology, Conceptualization.

## Conflicts of Interest

The authors declare no conflicts of interest.

## Data Availability

The data that support the findings of this study are available from the corresponding author upon reasonable request.
